# Correlation of PMN elastase and PMN elastase-to-neutrophil ratio with disease activity in patients with myositis

**DOI:** 10.1186/s12967-019-02176-z

**Published:** 2019-12-16

**Authors:** Siyu Wu, Wanchan Peng, Yunli Zhang, Jingjing Guo, Jinfang Fu, Wei Wang

**Affiliations:** Department of Clinical Laboratory, Xiangya Hospital, Central South University, 87 Xiangya Road, Changsha, Hunan People’s Republic of China

**Keywords:** PMN elastase, PMN elastase-to-neutrophil ratio, Idiopathic inflammatory myopathies, Disease activity

## Abstract

**Background:**

Polymorphonuclear (PMN) elastase plays an important role in a variety of inflammatory disorders. Our aim was to analyse PMN elastase in idiopathic inflammatory myopathies (IIMs) and its association with disease activity.

**Methods:**

PMN elastase levels were measured using enzyme-linked immunosorbent assay in serum samples obtained from 74 patients with myositis (58 with dermatomyositis [DM] and 16 with polymyositis [PM]) and 22 healthy controls. Receiver operating characteristic (ROC) curve analysis was performed to evaluate the discriminant capacity of PMN elastase level and PMN elastase-to-neutrophil ratio (ENR) in patients with active and remission myositis. The association of serum PMN elastase level and ENR with disease variables was evaluated in patients with IIMs. The disease specificity of PMN elastase level and ENR was further examined in 60 patients with other systemic autoimmune diseases.

**Results:**

PMN elastase level and ENR were significantly higher in patients with active IIMs, DM, and PM than in patients with remission. ROC curve analysis revealed that PMN elastase level and ENR both outperformed creatine kinase (CK), the currently used laboratory marker, and strongly discriminated patients with active disease and those with remission of IIMs, DM, and PM (area under the ROC curve [AUC] 0.9, 0.9, and 0.88 for PMN elastase; AUC 0.96, 0.96, and 1.0 for ENR; AUC 0.72, 0.70, and 0.80 for CK, respectively). PMN elastase level and ENR were positively correlated with myositis disease activity assessment, CK, lactate dehydrogenase, aspartate aminotransferase, alanine aminotransferase, C-reactive protein, and erythrocyte sedimentation rate. PMN elastase level and ENR were higher in the anti-PM-Scl positive myositis group than those in the anti-PM-Scl negative myositis group. Nevertheless, PMN elastase was not a specific disease marker for IIMs when compared with other autoimmune diseases.

**Conclusions:**

PMN elastase, particularly ENR, were significantly correlated with disease activity and could serve as useful biochemical markers for evaluating the disease activity of patients with IIMs. Thus, they are potentially helpful in monitoring disease progression and guiding treatment.

## Background

Idiopathic inflammatory myopathies (IIMs), including dermatomyositis (DM), polymyositis (PM), inclusion body myositis, and immune-mediated necrotising myopathy, which have been recently distinguished [1–3], are rare systemic autoimmune diseases. They are characterised by chronic proximal muscle inflammation and weakness, and/or skin rash. The serological features include a high titration of serum autoantibodies and muscle enzymes, and the histological features are infiltration of mononuclear cells and inflammatory cells in the skeletal muscle tissue [1, 4]. Inflammatory cell infiltration in muscle tissue may contribute to the muscle damage and dysfunction by releasing cytokines, cytotoxic molecules, or proteinase, leading to disease progression.

Disease activity of IIMs is currently assessed using muscle enzyme testing and clinical evaluation. Muscle enzyme testing includes serum creatine kinase (CK), aspartate aminotransferase (AST), alanine aminotransferase (ALT) and lactate dehydrogenase (LDH) activity assessment and has been shown to moderately correlate with disease activity in IIMs [5, 6]. However, clinical evaluation depends on the experience of health care professionals and patient collaboration [7, 8]. Thus, identifying objective tools or biomarkers is urgent for monitoring IIM disease activity. Recently, longitudinal cohort and multi-cohort validation studies have revealed that galectin-9 and CXCL-10 are sensitive and reliable biomarkers for disease activity in juvenile DM [9, 10]. In adult patients with myositis, several studies revealed that S100A11 [11], serum-soluble TRAIL [12], YKL-40 [13], and anti-EJ [14] are correlated with disease activity, but there is a lack of further research on their diagnostic value. Hence, objective biomarkers for monitoring disease activity in adult patients with myositis need further investigation.

Polymorphonuclear (PMN) leukocytes or neutrophils are inflammatory cells that play an important role as primary defence cells in the inflammatory response against invading pathogens or damaged tissue, by using proteinases. One of these proteinases is PMN elastase, which is localised in the azurophilic granules. The activity of PMN elastase is regulated by proteinase inhibitors, such as α1-proteinase inhibitor [15]. An overwhelming release of PMN elastase, which exceeds the inhibitory potential of the proteinase inhibitor, can lead to active PMN elastase persisting. In combination with simultaneously produced oxidants (O_2_-radicals, H_2_O_2_, OH-radicals), this can cause local tissue injury. Previous studies showed that PMN elastase plays an important role in a variety of inflammatory disorders [16–19]. Most studies have demonstrated a clear correlation between PMN elastase and clinical course of the disease, and PMN elastase may be a biochemical marker reflecting the intensity of inflammation in patients with inflammatory diseases [20–22]. Moreover, studies on PMN elastase and IIMs showed that neutrophil serine proteinases, including cathepsin G, PMN elastase, and proteinase 3, are upregulated in patients with IIMs and increase the permeability of vascular endothelial cells to permit infiltration of inflammatory cells into the muscle [23, 24]. However, the role of PMN elastase in evaluating the disease activity of patients with IIM remains unknown and needs further investigation. Thus, the major aim of this study was to evaluate serum PMN elastase levels in patients with IIMs and other autoimmune diseases, with an emphasis on investigating the role of PMN elastase to distinguish between patients with active IIMs and those in remission.

## Methods

### Human subjects

The study group comprised 74 patients with myositis (58 with DM, 16 with PM). Patients with PM and DM fulfilled the Bohan and Peter diagnostic criteria [25, 26] and had no other systemic autoimmune diseases, infections, or major illnesses. Disease activity was assessed using the myositis disease activity assessment (MYOACT), established by the International Myositis Assessment and Clinical Studies group [27]. This tool was proved to be reliable for evaluating disease activity in Chinese patients with DM or PM [28]. Active disease was defined by clinicians based on clinical criteria, including typical skin manifestations, muscle involvement, and/or an increase in muscle enzyme levels. The patients with myositis in this study were also suffering from arthritis (30%), dysphagia (2.7%), or ulceration (2.7%). In addition, 43% of the patients with myositis were diagnosed with interstitial lung disease (ILD) and 2.7% with cardiac involvement. ILD was identified using high-resolution computed tomography.

The control group included healthy controls (HCs, n = 22) matched for age and sex with the patients. To determine the disease specificity of the biomarkers, different disease controls were added to the study: systemic lupus erythematosus (SLE, n = 20), systemic sclerosis (SSc, n = 20), and rheumatoid arthritis (RA, n = 20). Patients with RA were diagnosed according to the American College of Rheumatology/European League against Rheumatism classification criteria. Diagnosis of SLE and SSc were performed based on the American College of Rheumatology criteria. Active disease was assessed mainly by clinicians and defined as having an SLE disease activity index score ≥ 4 for SLE [29], the European Scleroderma Trails and Research group activity index score > 2.5 for SSc [30], and a disease activity score 28 ≥ 3.2 for RA [31].

Informed written consent was obtained from all subjects, and the study was approved by the Ethics Committee of Xiangya Hospital, Central South University, where the study was performed.

### Laboratory analysis

The following data for patients with IIMs were additionally collected: CK, LDH, ALT, AST, erythrocyte sedimentation rate (ESR), C-reactive protein (CRP), complement fractions (C3 and C4), neutrophil and lymphocyte counts, and the ratio of neutrophil to lymphocyte (NLR). These were all analysed using routine laboratory techniques within 24 h after enrolment. Antinuclear antibody (ANA) was detected by indirect immunofluorescence using Hep-2 cells (Euroimmun, Lübeck, Germany). Myositis-specific and -associated autoantibodies were detected using immunoblotting (Euroimmun, Lübeck, Germany). The demographic data for the disease groups are described in Table 1.

**Table 1:** Clinical characteristics of the study subjects

	IIMs	DM	PM	SLE	SSc	RA
Number	74	58	16	20	20	20
Gender (female/male)	55/19	41/17	14/2	17/3	12/8	18/2
Age (years)	50 (20–75)	50 (20–75)	51 (32–69)	41 (14–62)	57 (14–73)	58 (44–70)
Disease durations (years)	0.8 (0.1–12)	0.8 (0.1–12)	0.7 (0.1–4)	3.5 (0.1–15)	2 (0.7–7)	1 (0.1–10)
Active/remission (number)	44/30	35/23	9/7	10/10	10/10	10/10
MYOACT	1.4 (0.7–4.2)	1.4 (0.7–4.2)	1.4 (0.7–2.6)	–	–	–
Muscle enzymes						
CK, uKat/L	59 (12.5–6621)	58 (12.5–6621)	65.1 (21.3–5890.8)	–	–	–
AST, uKat/L	30.8 (11.2–420.1)	30.8 (12.4–420.1)	29.8 (11.2–227.6)	21.7 (13.5–68.9)	25.1 (13.5–66.3)	17.3 (9.6–45.2)
ALT, uKat/L	29.9 (6.2–201.8)	31.7 (10.4–201.8)	25.7 (6.2–139)	19 (9.6–152.2)	14.3 (3.6–72.3)	13.4 (4.9–59.9)
LDH, uKat/L	284 (137–1267)	282.5 (141–1267)	286 (137–767)	–	–	–
Inflammation marker						
CRP, mg/L	3.2 (1–165)	3.8 (1.1–165)	2.5 (1–7.1)	5.1 (1.3–242)	4.1 (1–43.2)	14.1 (1.5–86.5)
ESR, mm/h	49 (4–120)	53 (4–120)	30 (6–85)	55 (2–120)	54.5 (2–114)	92.5 (33–120)
C4, mg/L	203.5 (39.6–454)	222 (39.6–454)	185 (102–437)	153 (23.2–365)	188 (108–375)	208 (117–318)
C3, mg/L	836 (415–1690)	826 (415–1690)	803 (566–955)	704 (81–1210)	795 (411–1410)	1010 (817–1220)
Neutrophil, × 10^9^/L	4.5 (1.4–15.1)	5.2 (1.4–12.4)	4.7 (1.5–15.1)	4.8 (0.7–11.8)	4.4 (1.8–11.5)	4.5 (1.9–8.6)
Lymphocyte, × 10^9^/L	1.4 (0.1–4.1)	1.3 (0.1–4.1)	1.4 (0.3–3.7)	1.4 (0.1–2.8)	1.3 (0.6–2.6)	1.6 (0.9–3.1)
NLR	3.7 (0.7–23)	4.4 (1.3–23)	3.9 (0.8–14)	3.6 (1.2–31)	3.3 (2.1–11.5)	3.0 (1.4–7.9)

Data are presented as number of patients or median (min–max)

*MYOACT* myositis disease activity assessment, *CK* creatine phosphokinase, *AST* aspartate aminotransferase, *ALT* alanine aminotransferase, *LD* lactate dehydrogenase, *CRP* C-reactive protein, *ESR* erythrocyte sedimentation rate, *C4* complement fraction 4, *C3* complement fraction 3, *NLR* the ratio of neutrophil count to lymphocyte count

### Measurement of serum PMN elastase levels

Serum samples were stored at − 20 °C until analysis. Serum levels of human PMN elastase were measured using a commercially available enzyme-linked immunosorbent assay kit (Abcam, Cambridge, MA). Standards and patient samples were analysed in duplicates according to the manufacturer’s instructions.

### Statistical analysis

Data were tested for normality using Kolmogorov–Smirnov test in the total sample and within each group of patients (DM and PM). Differences between groups were assessed with the Mann–Whitney U test. Since most of the variables were not normally distributed, data are shown as medians (min–max) or medians (interquartile range). The correlations between variables were evaluated using Spearman’s rank correlation. Receiver operating characteristic (ROC) curves were used to evaluate the significance of PMN elastase levels to distinguish between myositis patients with active disease and those in remission. The Youden index was calculated as sensitivity + specificity − 1. The best critical point was selected as the largest tangential point of the Youden index. A P value < 0.05 was used to indicate a statistically significant result. Statistical analysis was performed using SPSS 20.0 (IBM SPSS Statistics, IBM Corporation, Chicago, IL, USA) or Prism software v. 5.0 (GraphPad Software, Inc., San Diego, CA).

## Results

### Serum levels of PMN elastase in patients with myositis and controls

**Fig. 1:**
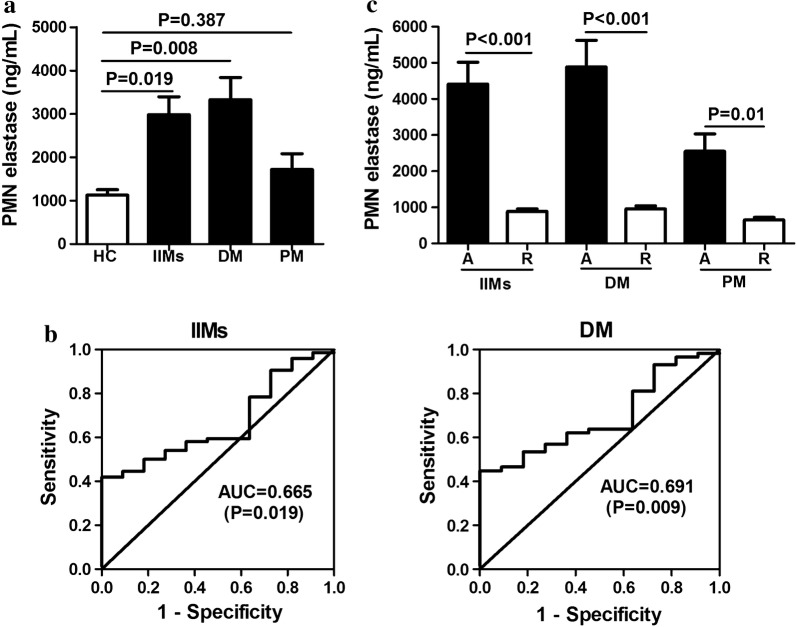
Serum levels of polymorphonuclear (PMN) elastase in patients with myositis. PMN elastase levels are elevated in patients with idiopathic inflammatory myopathies (IIMs) and dermatomyositis (DM), but not in those with polymyositis (PM) when compared with healthy controls (HCs) (**a**). Area under receiver operating characteristic (ROC) curve analysis was used to assess the ability of PMN elastase to distinguish among IIMs, DM, and HCs (**b**). The reference curve is also shown. PMN elastase levels were significantly higher in patients with IIMs, DM, and PM with active disease [A] than those in remission [R] (**c**)

The concentrations of PMN elastase in patients with myositis and HCs are shown in Fig. 1a. PMN elastase levels in patients with IIMs were particularly upregulated compared with those in HCs (1521.8 (787.4–3820.5) vs 1193.6 (518.8–1513.9), ng/mL, median (interquartile range); P = 0.019). After dividing the patients with IIMs into subgroups, only those with DM showed significantly higher elevation of PMN elastase when compared with HCs (1569.6 (871.1–4676.0) vs 1193.6 (518.8–1513.9), ng/mL, P = 0.008; Fig. 1a). No significant difference was found between patients with PM and HCs (999.7 (581.4–3204.5) vs 1193.6 (518.8–1513.9), ng/mL, P = 0.387; Fig. 1a).

The predictive values of PMN elastase in patients with IIMs and DM versus those in HCs were studied using univariate ROC analysis. The univariate areas under the curve (AUC) for PMN elastase were 0.665 (95% CI 0.55–0.78) for discriminating between patients with IIMs and HCs (P = 0.019; Fig. 1b) and 0.691 (95% CI 0.58–0.81) for discriminating between patients with DM and HCs (P = 0.009; Fig. 1b).

### Correlation of PMN elastase levels with disease activity

To investigate the correlation of PMN elastase levels with disease activity, we initially compared IIM patients with active disease and those in remission. PMN elastase levels were significantly higher in patients with active disease than those in remission (3382.9 (1722.5–5821.6) vs 799.8 (556.7–1269.1), ng/mL, P < 0.001); Fig. 1c). After dividing the total patients into DM and PM, both patients with active DM and PM showed higher PMN elastase levels than those in remission (3413.5 (1891.9–5860.0) vs 875.2 (695.2–1425.0), and 2612.6 (1258.1–3705.9) vs 619.8 (449.8–806.3), ng/mL, P < 0.001 and P = 0.01; Fig. 1c).

**Fig. 2:**
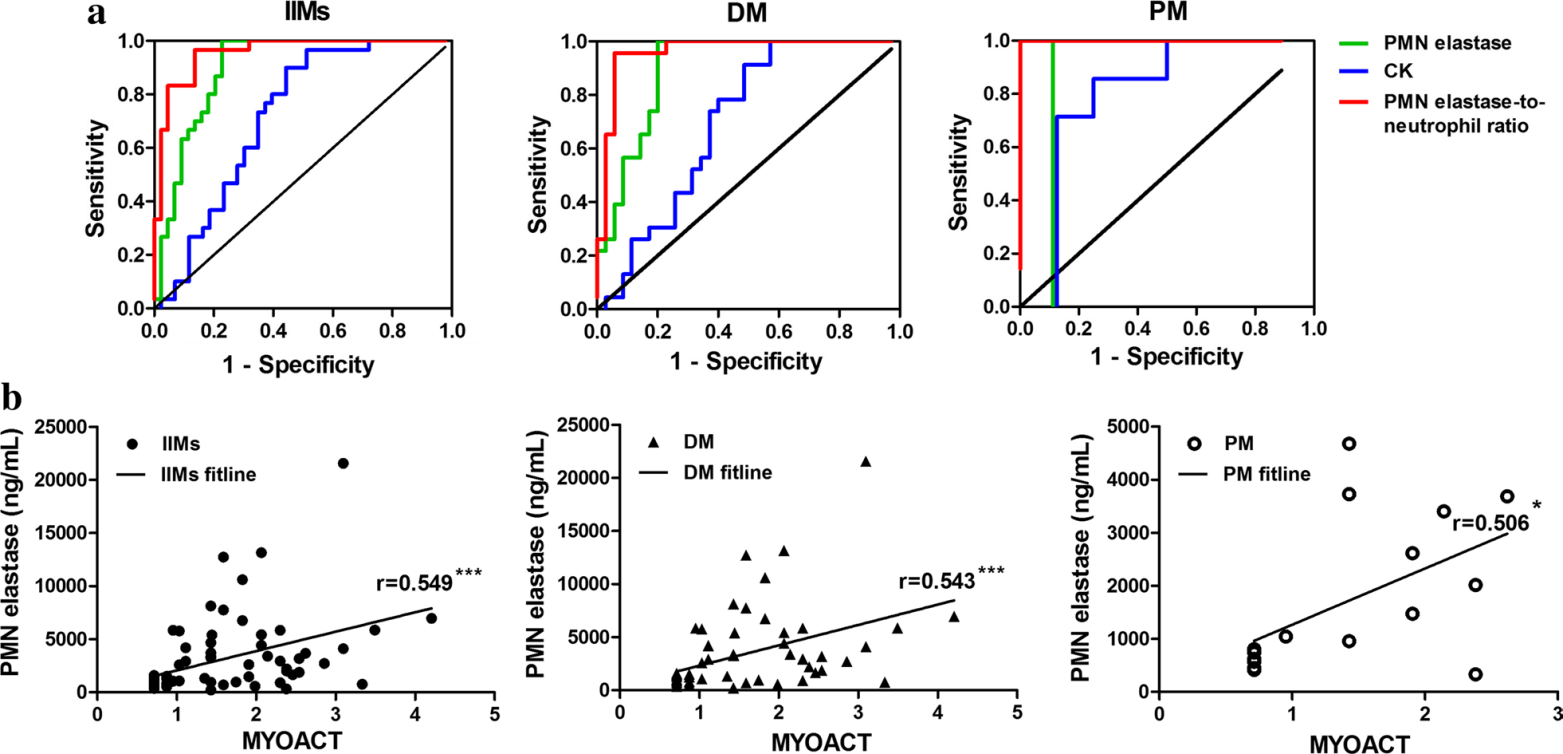
Correlation of polymorphonuclear (PMN) elastase levels with disease activity. Area under receiver operating characteristic (ROC) curve analysis was used to assess the ability of PMN elastase, creatine kinase (CK), and PMN elastase-to-neutrophil ratio (ENR) to distinguish between patients with active idiopathic inflammatory myopathies (IIMs), dermatomyositis (DM), and polymyositis (PM) and those in remission (**a**). The reference curve is also shown. The correlations between PMN elastase levels and myositis disease activity assessment (MYOACT) were assessed using Spearman’s rank correlation coefficients (**b**). Statistical significance is indicated by asterisks (*P < 0.5, ***P < 0.001)

To further assess the discriminative power of PMN elastase for active disease and remission, univariate ROC analysis was performed. Comparing active disease and remission, PMN elastase had an AUC of 0.90 for IIMs, 0.90 for DM, and 0.88 for PM (Fig. 2a and Table 2). Based on the coordinates of the ROC curves, the cut-off values of PMN elastase were 1632.4 ng/mL for IIMs, 1779.0 ng/mL for DM, and 999.7 ng/mL for PM, which resulted in a sensitivity of 79%, 80%, and 80% and a specificity of 100% for IIMs, DM and PM, respectively (Table 2). Moreover, PMN elastase performed better than the current standard laboratory marker CK in patients with myositis (AUC of CK: 0.72 in IIMs, 0.70 in DM, 0.80 in PM).

**Table 2:** AUC, PPV, NPV, sensitivity and specificity for PMN elastae in patients with myositis

	IIMs			DM			PM		
	PMN elastase	CK	ENR	PMN elastase	CK	ENR	PMN elastase	CK	ENR
AUC (95% CI)	0.90*** (0.83–0.97)	0.72** (0.61–0.84)	0.96*** (0.92–1.0)	0.90*** (0.82–0.98)	0.70* (0.57–0.83)	0.96*** (0.91–1.0)	0.88* (0.65–1.0)	0.80* (0.56–1.0)	1.0** (1.0–1.0)
Cut off value (ng/mL)	1632.4	84.8	329.0	1632.4	107.9	329.0	1632.4	107.9	329.0
PPV	1	0.89	0.98	1	1	0.98	1	1	1
NPV	0.77	0.59	0.85	0.68	0.44	0.89	0.78	0.58	1
Sensitivity (%)	79	56	88	80	42.9	94	80	43	94
Specificity (%)	100	90	97	100	100	96	100	100	96

*AUC* area under the curve, *PPV* positive predictive value, *NPV* negative predictive value; *95% CI* 95% confidence interval, *ENR* PMN elastase-to-netrophil ratio

* P < 0.05 for the area under the curve; **P < 0.01 for the area under the curve; ***P < 0.001 for the area under the curve

Moreover, we investigated the correlation of PMN elastase level with disease activity scoring by using the MYOACT tool. Notably, the PMN elastase level was positively correlated with MYOACT, with r values of 0.549 in IIMs (P < 0.001; Fig. 2b), 0.543 in DM (P < 0.001; Fig. 2b), and 0.506 in PM (P < 0.05; Fig. 2b). Overall, these data emphasise the utility of PMN elastase as a potential biomarker for disease monitoring.

### PMN elastase-to-neutrophil ratio (ENR) value to distinguish active disease and remission

**Fig. 3:**
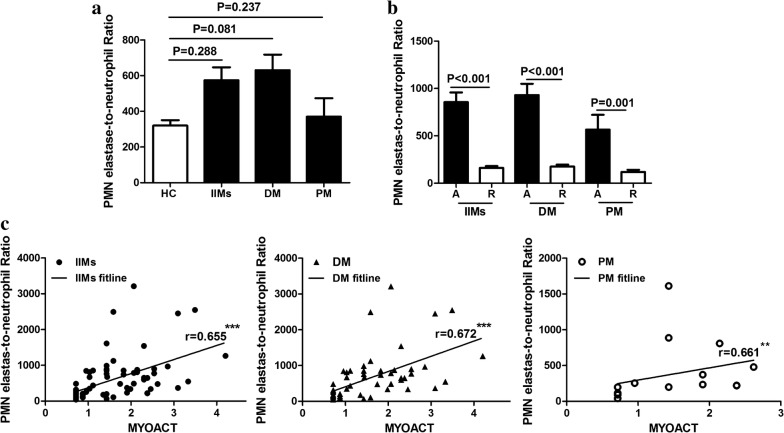
Levels of polymorphonuclear (PMN) elastase-to-neutrophil ratio (ENR) in patients with myositis and correlation of ENR with disease activity. ENR showed no difference in patients with idiopathic inflammatory myopathies (IIMs), dermatomyositis (DM), and polymyositis (PM) when compared with HCs (**a**). ENR was significantly higher in patients with IIMs, DM, and PM with active disease [A] than those in remission [R] (**b**). ENR showed moderately positive correlations with myositis disease activity assessment (MYOACT) in patients with IIMs, DM, and PM (**c**). Statistical significance is indicated by asterisks (**P < 0.01, ***P < 0.001)

Considering the diagnostic value of PMN elastase in distinguishing myositis patients with active disease and those with remission and the correlation of PMN elastase with neutrophil count (Table 3), we further investigated whether ENR, which is the ratio of serum PMN elastase level to serum neutrophil count, was correlated with disease activity. Results showed that ENR did not differ in patients with IIMs, DM and PM when compared with HCs (all P > 0.05; Fig. 3a). In patients with IIMs with active disease, ENR was significantly higher than those in remission (751.5 (389.4–897.5) vs 146.3 (93.6–199.8), P < 0.001; Fig. 3b). After dividing the patients with IIMs into patients with DM and PM, both patient subgroups with active disease showed higher ENR than those in remission (776.1 (487.0–967.9) vs 151.8 (100.2–208.8), and 373.2 (227.8–848.5) vs 97.8 (91.7–196.7), P < 0.001 and P = 0.001; Fig. 3b). ROC analysis was performed, and the ENR to distinguish active disease and remission had an AUC of 0.96 for IIMs, 0.96 for DM, and 1.0 for PM (Table 2). The sensitivity and specificity of ENR for active disease in IIMs were 88% and 97%, and those in DM and PM were both 94% and 96%, respectively (Table 2). Moreover, ENR was positively correlated with MYOACT, with r values of 0.655 in IIMs (P < 0.001; Fig. 3c), 0.672 in DM (P < 0.001; Fig. 3c), and 0.661 in PM (P < 0.01; Fig. 3c).

**Table 3:** Association of PMN elastase levels and ENR with laboratory parameters of patients with myositis

	PMN elastase			ENR		
	IIMs	DM	PM	IIMs	DM	PM
Muscle enzymes						
CK	0.308**	0.339**	0.229	0.289**	0.332**	
LDH	0.366**	0.386**	0.211	0.287*	0.295*	0.307
ALT	0.437***	0.406***	0.288	0.343**	0.296*	0.251
AST	0.438***	0.454***	0.23	0.419***	0.442***	0.197
Inflammation marker						
CRP	0.35**	0.346*	0.194	0.329**	0.357**	0
ESR	0.315**	0.292**	0.146	0.362**	0.4**	0.006
C4	0.11	0.02	0.168	0.159	0.121	− 0.077
C3	− 0.023	− 0.064	0.214	0.01	0.017	− 0.068
Neutrophil	0.286**	0.339**	0.284	− 0.174	− 0.104	− 0.132
Lymphocyte	− 0.275	− 0.279	− 0.314	− 0.388	− 0.432***	− 0.241
NLR	0.443***	0.485**	0.361*	0.207*	0.291*	0.005

Data were analysed using the Spearman’s rank correlation

*ENR* PMN elastase-to-netrophil ratio, *CK* creatine phosphokinase, *AST* aspartate aminotransferase, *ALT* alanine aminotransferase, *LDH* lactate dehydrogenase, *CRP* C-reactive protein, *ESR* erythrocyte sedimentation rate, *C4* complement fraction 4, *C3* complement fraction 3, *NLR* the ratio of neutrophil count to lymphocyte count

### Correlation of PMN elastase and ENR with muscle enzymes and inflammatory markers

In patients with IIMs and DM, correlation analysis revealed that the serum PMN elastase level was positively associated with CK (r = 0.308 and 0.339, both P < 0.01), LDH (r = 0.366 and 0.386, both P < 0.01), ALT (r = 0.437 and 0.406, both P < 0.001), and AST (r = 0.438 and 0.454, both P < 0.001). Furthermore, positive associations were found between PMN elastase and CRP (r = 0.350 and 0.346, P < 0.01 and P < 0.05) and ESR levels (r = 0.315 and 0.292, both P < 0.01), neutrophil count (r = 0.286 and 0.339, both P < 0.01), and NLR (r = 0.443 and 0.485, P < 0.001 and P < 0.01). No correlations were found for PMN elastase with C4 and C3 levels or lymphocyte count. In patients with PM, the positive association was only found between PMN elastase and NLR (r = 0.361, P < 0.05).

We also observed correlations between ENR and CK (r = 0.289 and 0.332, both P < 0.01), LDH (r = 0.287 and 0.295, both P < 0.05), ALT (r = 0.343 and 0.296, P < 0.01 and P < 0.05), AST (r = 0.419 and 0.442, both P < 0.001), CRP (r = 0.329 and 0.357, both P < 0.01), ESR (r = 0.362 and 0.400, both P < 0.01), and NLR (r = 0.207 and 0.291, both P < 0.05) in patients with IIMs and DM. ENR showed a moderately negative correlation with lymphocyte count (r = − 0.432, P < 0.001) only in patients with DM. However, in patients with PM, no correlation was found between ENR and these indicators. Associations of PMN elastase levels and ENR with muscle enzymes and inflammation markers for each subgroup and all patients with myositis are listed in Table 3.

### Association of PMN elastase and ENR with autoantibodies in patients with myositis

In patients with IIMs, the prevalence of ANA, anti-Ro-52, anti-MDA5, anti-Jo-1, anti-TIF-1γ, anti-Mi-2, and anti-PM-Scl was 90%, 55.4%, 28.4%, 20.3%, 18.9%, 14.9%, and 10.8%, respectively. The prevalence of other autoantibodies was all less than 10%. We examined the difference in serum levels of PMN elastase and ENR between patents with and without elevation of an autoantibody. Our data showed that the serum levels of PMN elastase and ENR were higher in the anti-PM-Scl positive group than in the anti-PM-Scl negative group (5022.2 (2259.9–6684.8) vs 1445.8 (738.3–3404.8), ng/mL, P = 0.007, and 1127.4 (866.8–2224.2) vs 317.5 (146.4–692.6), P < 0.001) in patients with IIMs, which was also observed in patients with DM. By contrast, no significant differences were observed between other autoantibody positive and negative groups. The prevalence of autoantibodies and association of PMN elastase and ENR with autoantibodies for each subgroup and for all patients with myositis are summarised in Table 4.

**Table 4:** The associations of PMN elastase and ENR with autoantibodies in myositis patients

	IIMs		P value		DM		P value		PM		P value	
	P/N	%	PMN elastase	ENR	P/N	%	PMN elastase	ENR	P/N	%	PMN elastase	ENR
ANA	63/7	90.0	0.619	0.724	47/7	87.0	0.413	0.489	16/0	100	–	–
Anti-Ro-52	41/33	55.4	0.732	0.511	28/30	48.3	0.250	0.109	13/3	81.3	0.638	0.545
Anti-Jo-1	15/59	20.3	0.633	0.386	7/51	12.1	0.072	0.467	8/8	100	0.916	0.401
Anti-PM-Scl	8/66	10.8	0.007	0.001	8/50	16.0	0.013	0.001	0/16	0	–	–
Anti-MDA5	21/53	28.4	0.619	0.385	21/37	36.2	0.289	0.740	0/16	0	–	–
Anti-Mi-2	11/63	14.9	0.184	0.074	11/47	19.0	0.326	0.124	0/16	0	–	–
Anti-TIF-1γ	14/60	18.9	0.649	0.610	12/46	20.1	0.618	0.420	2/14	12.5	0.056	0.056
Anti-NXP2	4/70	5.4	0.830	0.793	3/55	5.2	0.352	0.562	1/15	6.3	0.159	0.159
Anti-PL7	4/70	5.4	0.056	0.054	4/54	6.9	0.08	0.07	0/16	0	–	–
Anti-Ku	2/72	2.7	0.334	0.443	1/57	1.7	0.179	0.16	1/15	6.3	0.745	0.745
Anti-EJ	4/70	5.4	0.304	0.189	1/57	1.7	0.128	0.269	3/13	18.8	0.737	0.946
Anti-SAE1	3/71	4.1	0.184	0.064	3/55	5.2	0.136	0.056	0/16	0	–	–
Anti-SRP	3/71	4.1	0.077	0.150	1/57	1.7	0.324	0.570	2/14	12.5	0.266	0.266

*ANA* antinuclear antibody, *ENR* PMN elastase-to-netrophil ratio

### Serum levels of PMN elastase and ENR in other autoimmune diseases

**Fig. 4:**
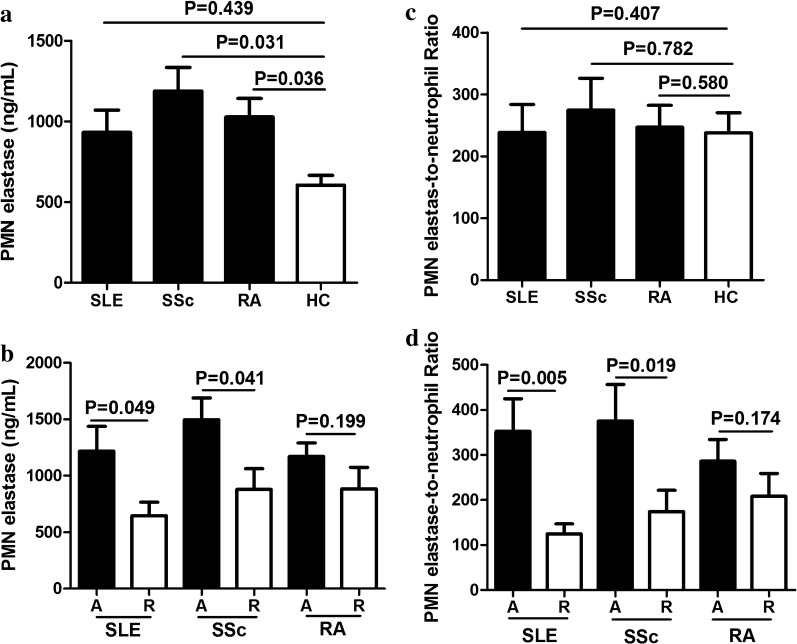
Polymorphonuclear (PMN) elastase level and PMN elastase-to-neutrophil ratio (ENR) in other autoimmune diseases. PMN elastase levels (**a**) and ENR (**c**) in patients with systemic lupus erythematosus (SLE), systemic sclerosis (SSc), and rheumatoid arthritis (RA) when compared with HCs. PMN elastase levels (**b**) and ENR (**d**) in patients with active SLE, SSc, and RA when compared with those in remission

To investigate whether PMN elastase and ENR were specifically elevated in myositis, PMN elastase levels and ENR in patients with SLE, SSc, and RA were measured. Results showed that the PMN elastase levels were slightly elevated in patients with RA and those with SSc (P = 0.036 and 0.031; Fig. 4a), but not in patients with SLE (P > 0.05; Fig. 4a). After dividing the patients into active and remission states, the PMN elastase levels were moderately higher in patients with active SLE and SSc than those in remission (P = 0.049 and 0.041; Fig. 4b).

Moreover, the ENR showed no differences in patients with SLE, SSc, and RA, when compared with HCs (all P > 0.05; Fig. 4c). However, after dividing the patients into active and remission states, the ENR in patients with active SLE and SSc was higher than those in remission (P = 0.005 and 0.019, respectively; Fig. 4d).

## Discussion

In this study, the level of PMN elastase was significantly increased in patients with IIMs and was positively correlated with clinical indicators, such as CK, LDH, ALT, AST, ESR, CRP, neutrophil count, and NLR. Importantly, PMN elastase strongly distinguished between IIM patients with active disease and those in remission. Moreover, we reported a new indicator, ENR. This indicator did not show increased levels in patients with IIMs when compared with HCs, but was superior to PMN elastase in evaluating the disease activity of patients with IIMs. This study is the first to focus on PMN elastase and ENR in distinguishing between active IIMs and IIMs in remission.

PMN elastase is a serine proteinase that is mainly stored in the azurophil granules of neutrophils [32]. When these cells are stimulated by microorganisms or other invading pathogens, PMN elastase is released into the extracellular space and exerts several effects, such as processing inflammatory mediators and combining with oxidants. Our results showed that PMN elastase levels were significantly higher in patients with IIMs and DM, which was consistent with the findings of the study carried out by Gao et al. [23]. In the total PM patient group, the PMN elastase level was not significantly altered when compared to that in healthy donors, but was significantly higher in those with active disease than those in remission. The reasons for the negative result between the PM patient group and healthy donors may due to the data variation between different PM patients, particularly those in remission, and the limited sample numbers of PM patients. We further compared the PMN elastase level between active PM patients and healthy donors; this showed that active PM patients have higher PMN elastase levels than healthy donors (P = 0.03, data not shown), which confirmed our supposition.

Previous studies also showed that in patients with RA [33] and SSc [34], the serum PMN elastase levels are elevated, and the activity of PMN elastase is increased in destructive joints in RA. These findings were similar to our results. However, no differences were found in patients with SLE, which may be due to the limited number of samples; thus, further investigation is warranted. These results suggest that during systemic inflammatory states or in autoimmune diseases, the serum PMN elastase level could increase. Interestingly, the PMN elastase levels were higher in patients with IIMs than those in patients with other autoimmune diseases in our study cohort. While the reasons for this difference are not clear, it might be related to the different pathogenetic mechanisms involved in the different diseases.

ENR, a new indicator reported first in this study, is the ratio of serum PMN elastase level to serum neutrophil count, representing the average level of elastase in each PMN cell. Plasma PMN elastase originates mainly from PMN cells and indicates the activity of PMN. Although the role of PMN elastase in inflammatory diseases has been partially investigated, data on ENR in inflammation are not available in the literature. We found that ENR did not increase in patients with IIMs, RA, SSc, and SLE when compared with HCs, but was significantly higher in patients with active IIM, SSc, and SLE than those in remission. The PMN elastase levels in patients with IIMs, RA, and SSc were higher than those in controls; PMN neutrophil counts in these diseases were correspondingly increased. PMN elastase levels were much higher in active disease than those in remission, but PMN neutrophil counts showed no difference between these two states. This indicates that PMN neutrophil counts are not reduced to basal levels in the remission state, and that PMN elastase levels not only correlate with PMN neutrophil counts, but also with neutrophil activity.

Our results show that both PMN elastase level and ENR may be useful biochemical markers for evaluating the disease activity of patients with IIMs. A study by Asim et al. revealed that in patients with psoriasis, PMN elastase may be a good marker for diagnosis and follow up of the disease activity, but the evidence in evaluating disease activity was limited [20]. Here, we propose this conclusion based on the following three points: first, PMN elastase level and ENR were significantly higher in patients with active IIMs than those in remission. Second, we found that serum PMN elastase level and ENR were positively correlated with MYOACT, CK, LDH, ALT, AST, CRP, and ESR. MYOACT is a tool used to assess myositis disease activity, and serum CK, LDH, ALT, and AST levels have been reported to have moderate correlation with muscle weakness and inflammation, which tend to indicate disease activity [5, 6, 35]. Serum levels of ESR and CRP are risk factors for ILD in DM/PM [36]. Third, the ROC curve analyses greatly supported our conclusions. PMN elastase level and ENR represent good bases for distinguishing between active and remission IIMs, with an AUC of 0.9 and 0.96. ENR was superior to PMN elastase in evaluating the disease activity of patients with IIMs. Furthermore, both biomarkers outperformed CK, which is commonly used as a laboratory marker for disease activity and is one of the current criteria for determining clinically inactive disease in juvenile DM [37, 38]. All these findings indicate that PMN elastase and ENR are potential markers for estimating disease activity in IIMs.

Current treatment guidelines recommend that immunosuppression is the traditional first-line treatment for patients with myositis, but treatment duration varies in different patients [39]. For example, some patients may benefit from shorter treatment duration; overtreatment with steroids can result in serious side effects. Hence, objective measurement of disease activity is crucial to determine the rate of medication tapering and to avoid both under- and overtreatment. Despite the novel and clinically relevant findings in this study, there are some limitations. The patients included in our study differed in severity and had different treatment options. Larger sample size and multi-cohort validation are needed. Thus, further studies should be performed to confirm our conclusion and promote this clinical application.

## Conclusion

This study is the first to show that PMN elastase and particularly ENR significantly correlate with disease activity. Thus, they might serve as biomarkers in patients with IIMs. Even in this small study cohort, the power of these markers to discriminate between patients with active disease and those in remission was highly significant, which emphasises their potential as biomarkers to monitor disease and guide treatment. Determination of the clinical value of PMN elastase and ENR is warranted in future prospective studies.

## Data Availability

All data generated or analysed during this study are included in this published article. If any additional information is required it may be obtained by request with the corresponding author.

## Abbreviations

ALT: alanine aminotransferase
ANA: antinuclear antibody
AST: aspartate aminotransferase
AUC: area under the ROC curve
C3: complement fractions 3
C4: complement fractions 4
CK: creatine kinase
CRP: C-reactive protein
DM: dermatomyositis
ENR: elastase-to-neutrophil ratio
ESR: erythrocyte sedimentation rate
HC: healthy control
IIM: idiopathic inflammatory myopathies
ILD: interstitial lung disease
LDH: lactate dehydrogenase
MYOACT: myositis disease activity assessment
NLR: neutrophil to lymphocyte ratio
PM: polymyositis
PMN: polymorphonuclear
RA: rheumatoid arthritis
ROC: receiver operating characteristic
SLE: systemic lupus erythematosus
SSc: systemic sclerosis
